# Supplementary material for: MiRNAs shape mouse age-independent tissue adaptation to spaceflight via ECM and developmental pathways
Source: Nat Commun. 2026 Feb 5;17:1387. doi: 10.1038/s41467-026-68737-1 (PMC12876965; doi:10.1038/s41467-026-68737-1)
Supplement: Supplementary file 1 — Supplementary Information [file 41467_2026_68737_MOESM1_ESM.pdf]

MiRNAs shape mouse age-independent tissue adaptation to spaceflight via ECM and developmental pathways

Supplementary Information

a b c

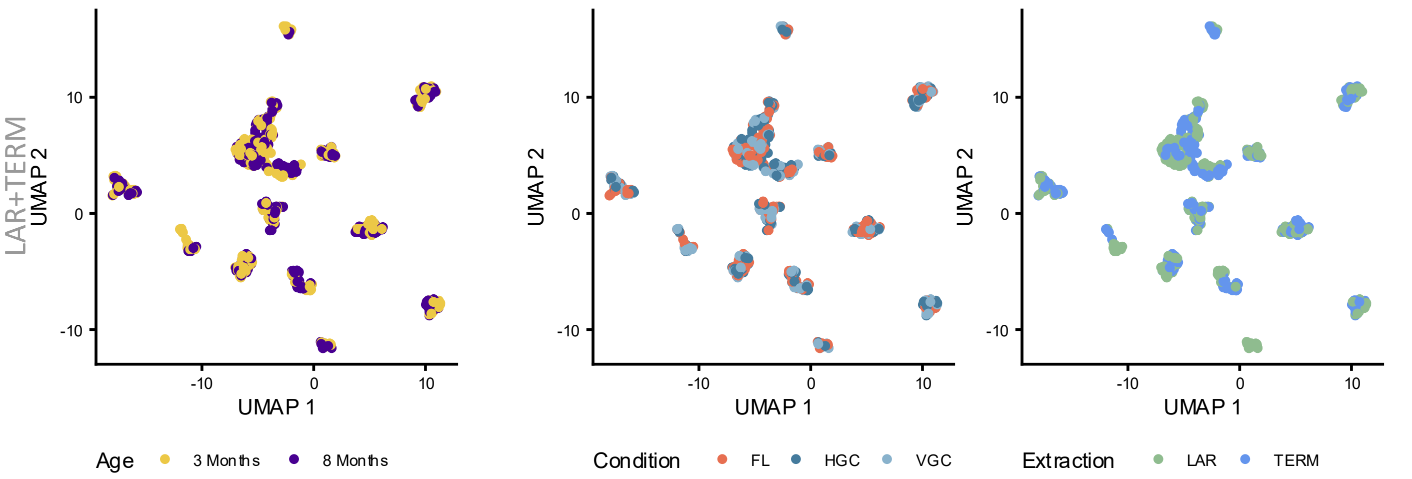

d

	TERM			LAR		
	FL	HGC	VGC	FL	HGC	VGC
Launch stress	X			X		
Exposure to spaceflight	X			X		
Double-density housing	X	X		X	X	
Return stress				X		
40d collection + slow freeze				X	X	X
21d collection + snap frozen	X	X	X			

e

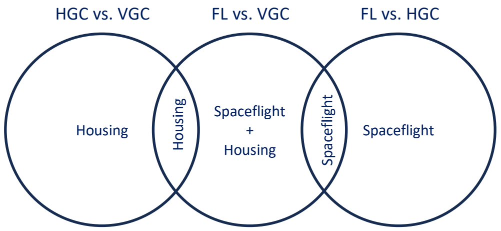

Supplementary Figure 1: Factors that contribute to the observed changes.

a-c. Two dimensional UMAP embedding colored by Age, Condition and Extraction method respectively. **d.** Factors that influence the different groups of the experiment.e.Venn diagram of effects captured by the overlap of each condition comparison. The comparison between FL and HGC primarily captures the deregulation coming from spaceflight, FL vs. VGC the deregulation coming from spaceflight and housing (FL vs. VGC) and HGC vs. VGC the deregulation coming from Housing.

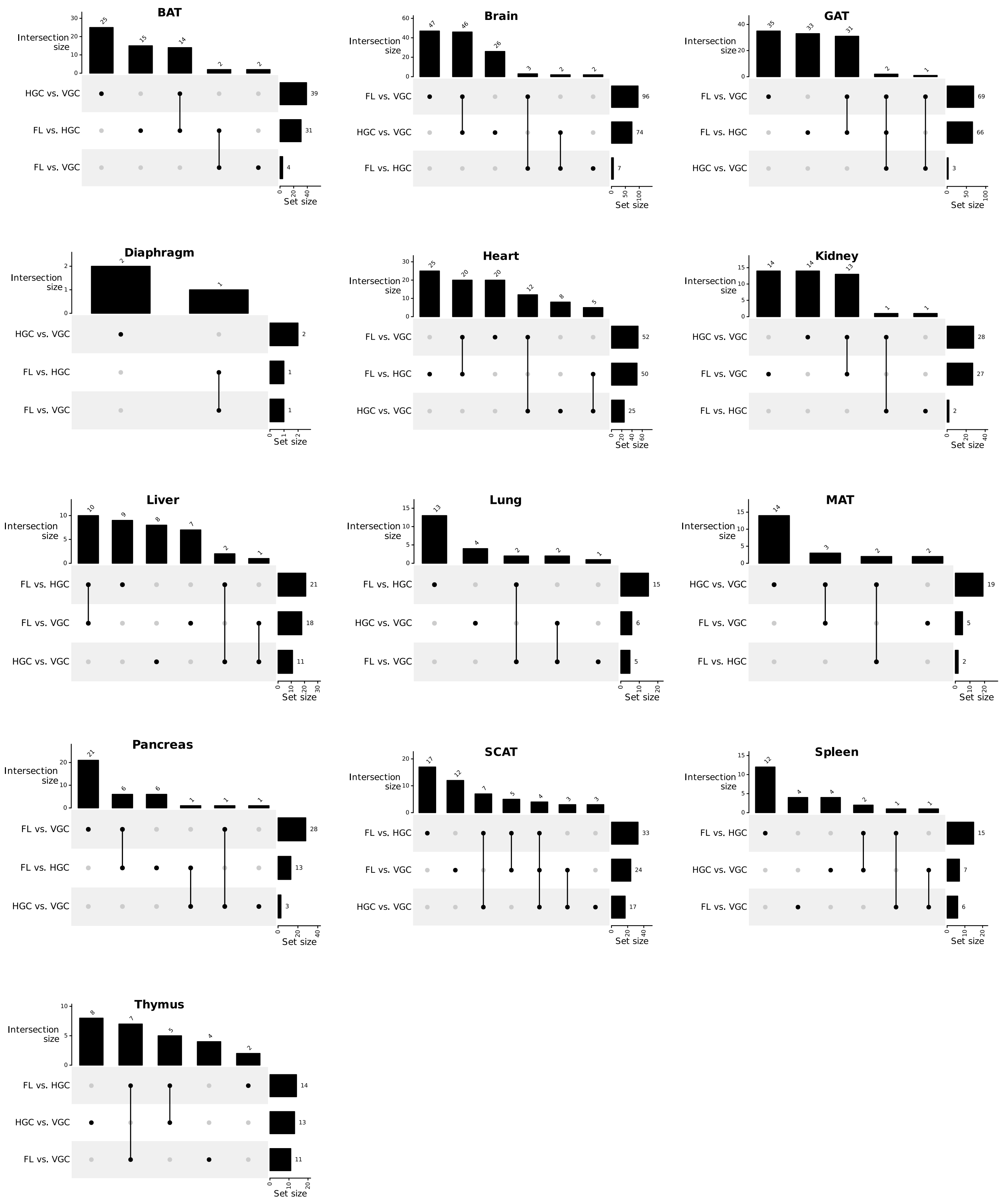

Supplementary Figure 2: UpSet plots showing the detailed overlaps from Figure 3a.
UpSet Plots per tissue showing the overlap between the deregulated miRNA under each condition comparison and overlap of conditions.

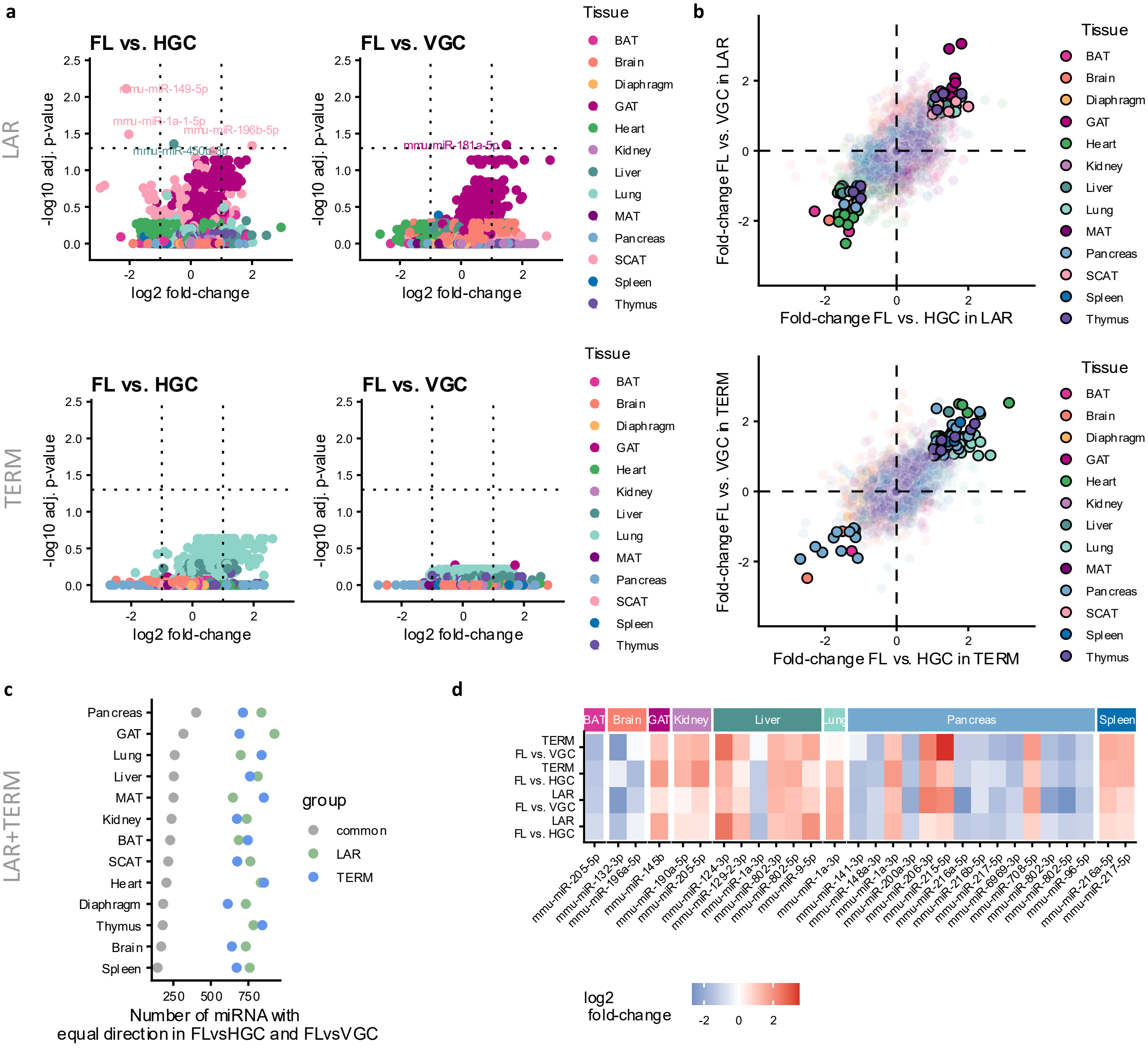

Supplementary Figure 3: Comparison of differentially expressed miRNA in LAR with TERM.
a. Deregulation of the miRNA in FL vs. HGC and FL vs. VGC in LAR and TERM. **b.** Comparison of the miRNA fold changes between FL vs. VGC and FL. vs. HGC in LAR and TERM groups respectively group. The opaque dots with dark border correspond to miRNA that are deregulated in both comparisons with matching fold change directions. **c.** MiRNAs with matching fold-change direction between FL vs. HGC and FL vs. VGC in each of LAR and TERM. The signal matching between the two extraction methods (and spaceflight exposure) corresponds to the “common” group. **d.** Overlapping miRNA from (e) that also show deregulation in at least one of the comparisons in LAR and in TERM.

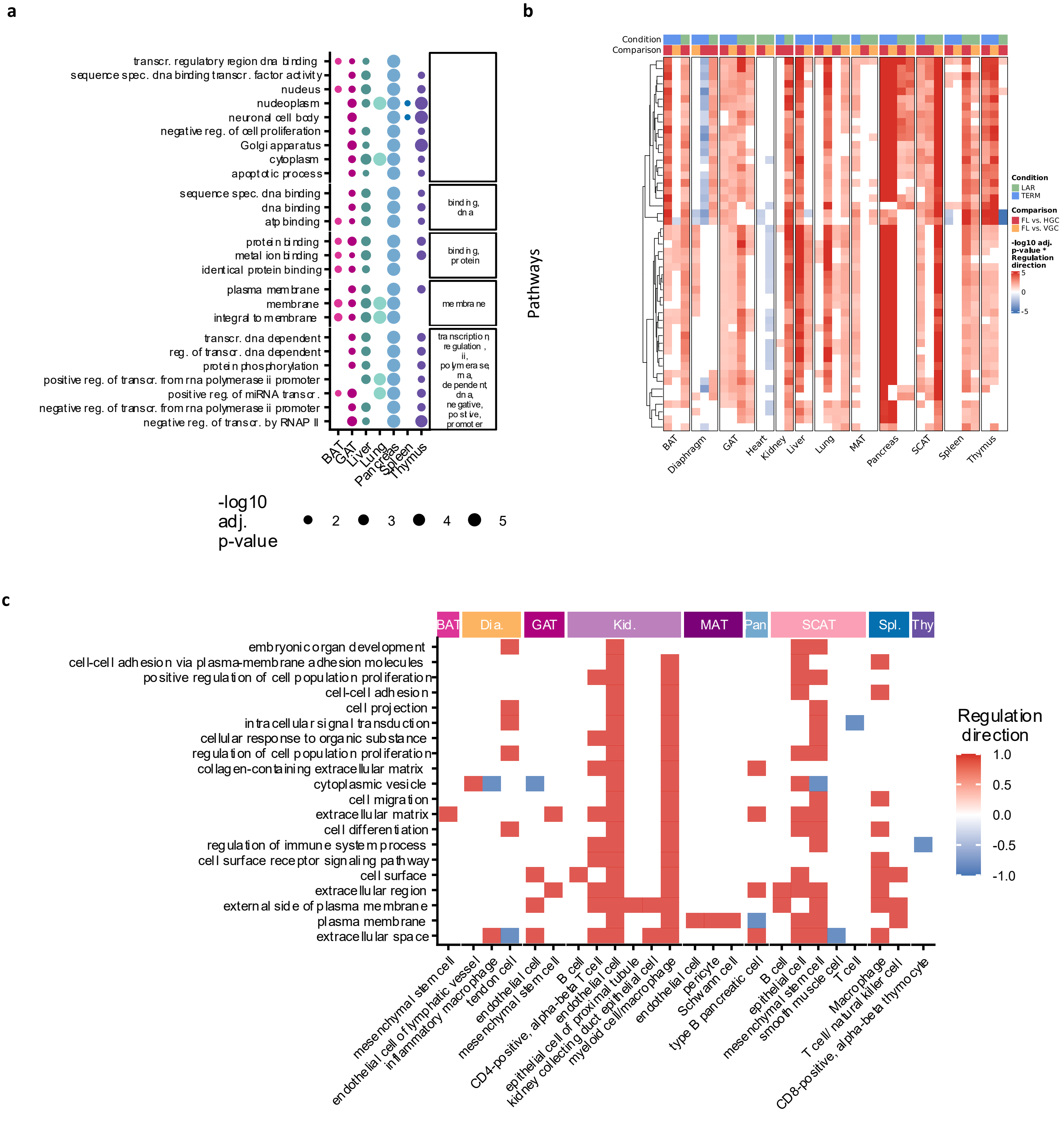

Supplementary Figure 4: **Pathway analysis of the miRNA in FL vs. HGC and FL vs. VGC.**
a. Top 25 pathways (GSEA, miEAA) in TERM as in Figure 3d using the miRNA lists sorted by fold-change. Pathways are clustered based on similarity in GO and labeled with the most frequent words. **b.** Enrichment and depletion of the top 25 pathways from Figure 3d and the top 25 from Supplementary Figure 4a in both LAR and TERM. **c.** Top 20 overlapping pathways in LAR between the miRNA (GSEA, miEAA) and single-cell mRNA (GSEA, genetrail) with matching direction of enrichment/depletion in at least one tissue.

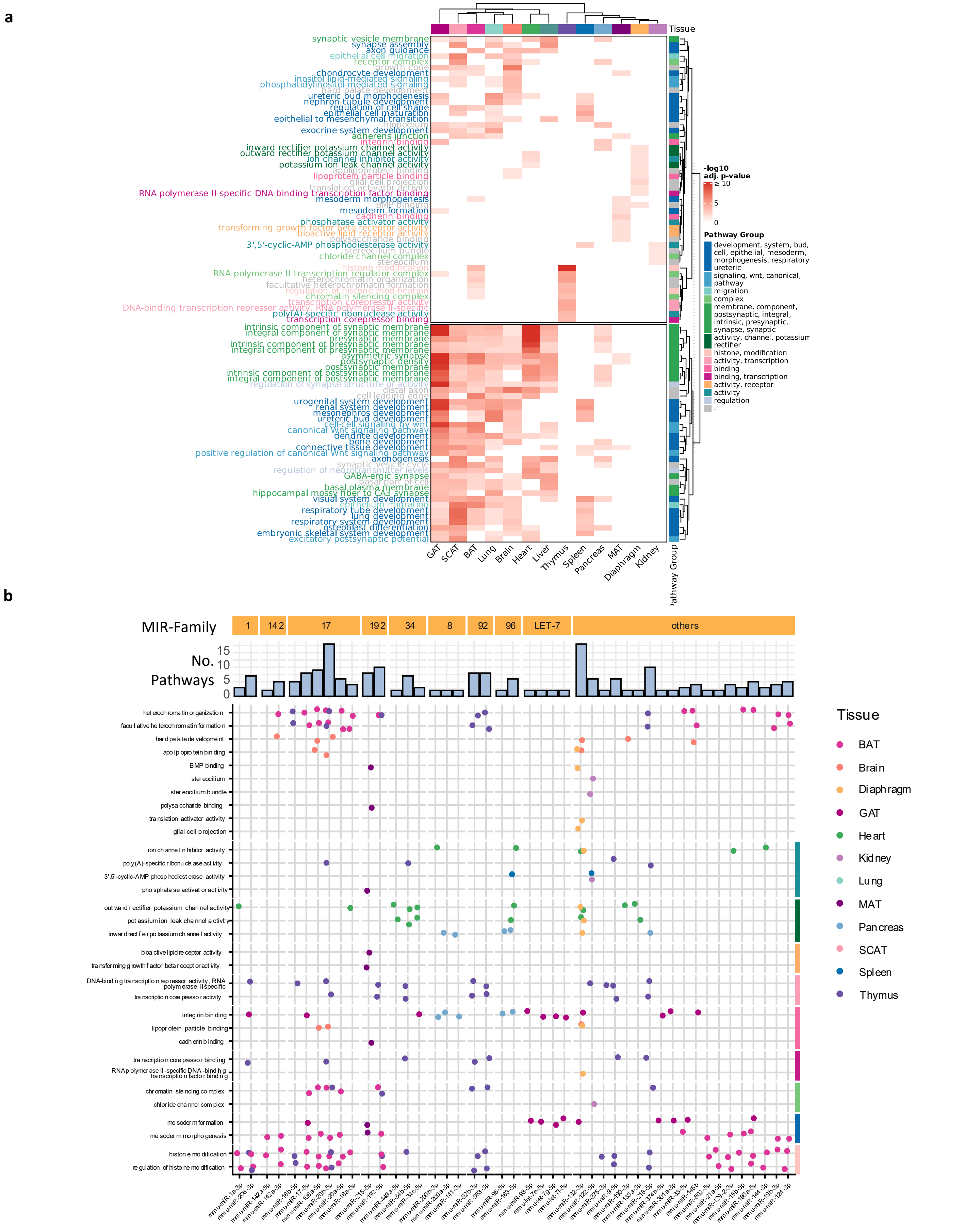

Supplementary Figure 5: **Detailed result of the Pathway analysis in mRNAs and miRNAs.**
a. Pathway analysis from Figure 4b in detail. **b.** The Cluster 1 pathways from Figure 4b that are only occurring in max. 2 tissues and the miRNAs that regulate the involved mRNAs and are part of more than one pathway show common miRNA deregulations.

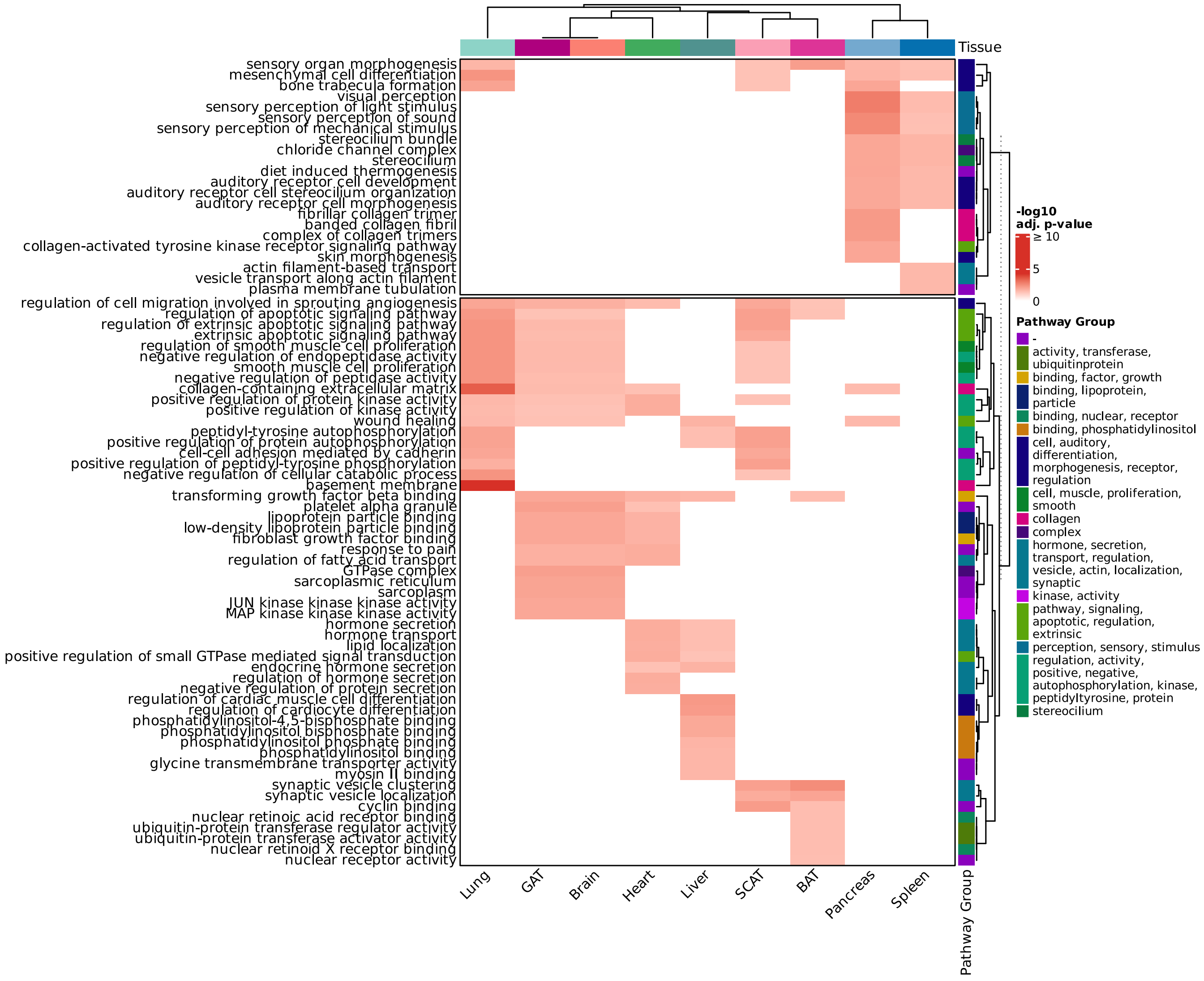

Supplementary Figure 6: Detailed result of the Pathway analysis using validated miRNA-mRNA interactions
Pathway analysis as in Figure 4b and Supplementary Figure 5a using only the experimentally validated miRNA-mRNA pairs (independent of the tissue they were validated in).

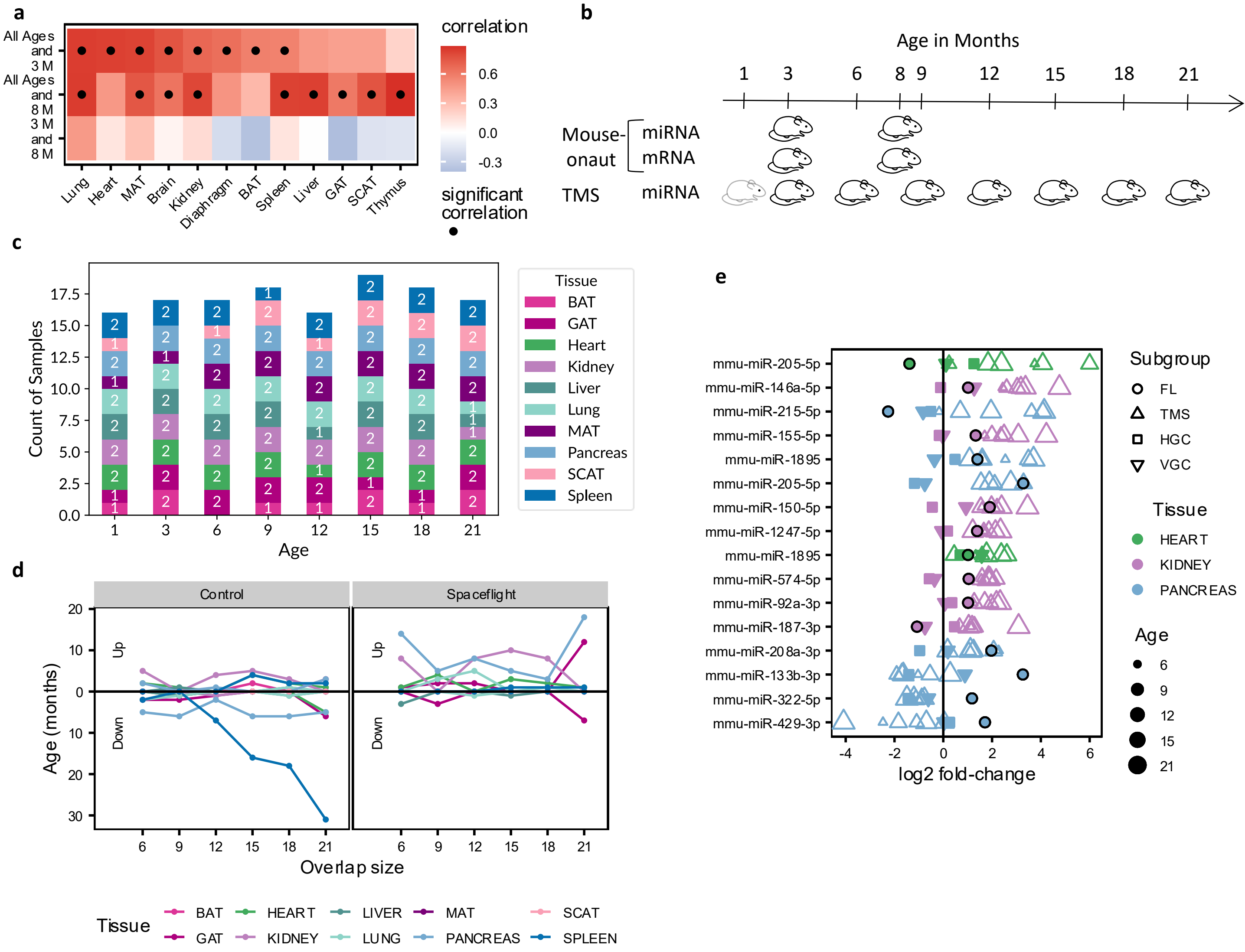

Supplementary Figure 7: Comparison of age-signatures with TERM and the TMS dataset

a. Correlation as in Figure 6c for the TERM group. **b.** TMS consists of 8 age-groups with male and female mice. For this comparison, we excluded the male mice and removed age 1 month to match the age of our younger age-group. The age-groups of the spaceflight mRNA (scRNA-seq) mice were matched with the age-groups in the miRNA mice. **c.** Sample counts for the different organs in TMS (only female). **d.** Number of common deregulated miRNAs in 3M vs. 8M in Space (FL) and Control (HGC) with the deregulated miRNAs in TMS in the comparison of the different ages (6,9,12,15,18 and 21 Months) with age 3M with matching direction of deregulation, split by upregulated miRNAs (Up) and downregulated miRNAs (Down). **e.** Comparison of log_2 fold changes in 8M vs. 3M for our FL, HGC and VGC groups with log_2 fold changes from the TMS project in the different age groups against the baseline (3M). miRNAs were selected if they were deregulated in more than 3 age groups.

a

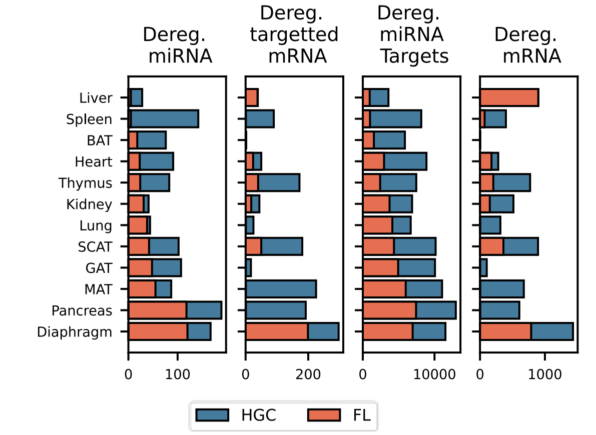

b

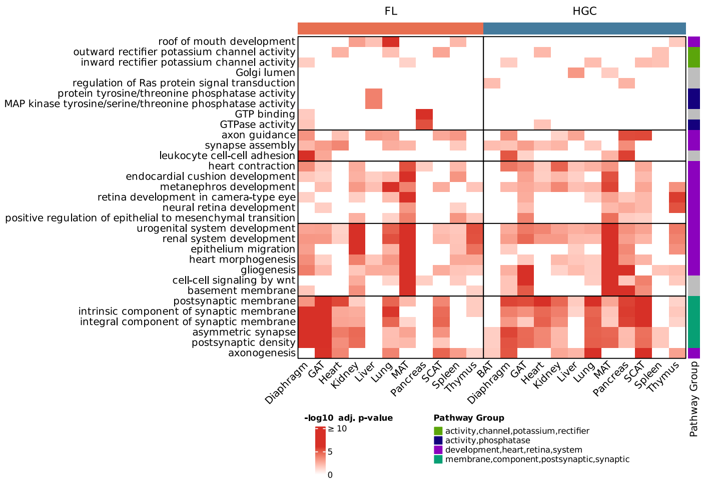

c

<table>
<tr><td></td><td></td><td colspan="4">Age dependent spaceflight effect filtering conditions</td></tr>
<tr><td>Baseline-Group</td><td>Test-Group</td><td colspan="2">FL 3M</td><td colspan="2">FL 8M</td></tr>
<tr><td>3M HGC</td><td>3M FL</td><td rowspan="2">↑
and
↓</td><td rowspan="2">↓
and
↑</td><td></td><td></td></tr>
<tr><td>3M FL</td><td>8M FL</td><td rowspan="2">↑
and
↑</td><td rowspan="2">↓
and
↓</td></tr>
<tr><td>8M HGC</td><td>8M FL</td><td></td><td></td></tr>
</table>

d

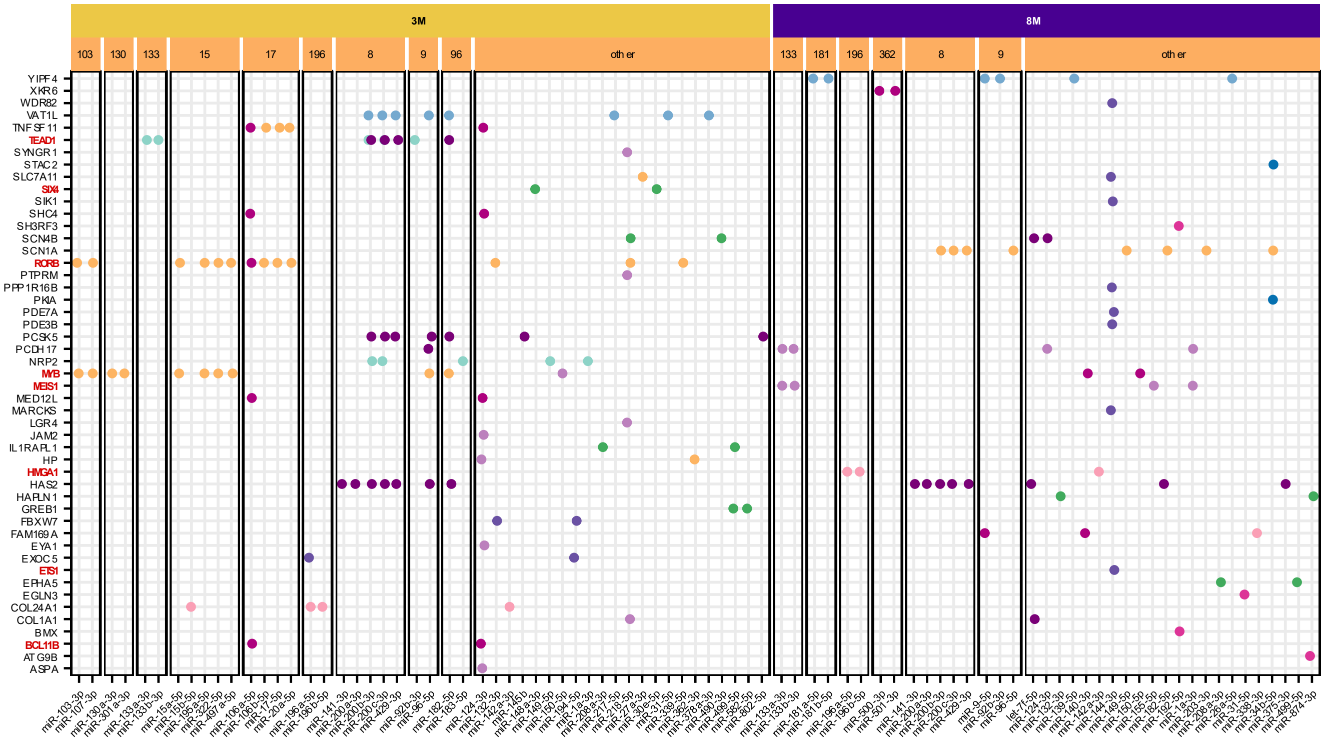

Supplementary Figure 8: mRNA-miRNA signatures driving age-specific signal

a. Comparison of deregulated miRNA and mRNA targets of these miRNA that were also found to be deregulated in the pseudo bulk single-cell mRNA arm for each tissue. The “deregulated targeted mRNA” shows the mRNA that were shown to be deregulated and are also the targets of deregulated miRNA. **b.** Pathway analysis of the mRNA-miRNA overlap (ORA, clusterProfiler; all GO databases) with the deregulated mRNAs that are targeted by miRNAs that are deregulated in 3M vs. 8M in HGC and show the same direction of fold-change in 3M vs. 8M in both HGC and in VGC. **c.** Schematic of comparison for filtering out spaceflight effects that are identical across age, leaving only spaceflight effects dependent on age. **d.** Detailed targeting of the miRNA-mRNA pairs included in Figure 7c. A dot represents targeting, while no dot means no targeting. Transcription factors are denoted by bold red text with an asterisk. Color of the dot represents the Tissue where the targeting is happening. MiRNAs are grouped according to age group in FL and the MIR family they are derived from. If in a timepoint, there is only 1 member from a MIR-family, they are put in the “other” family for space considerations.

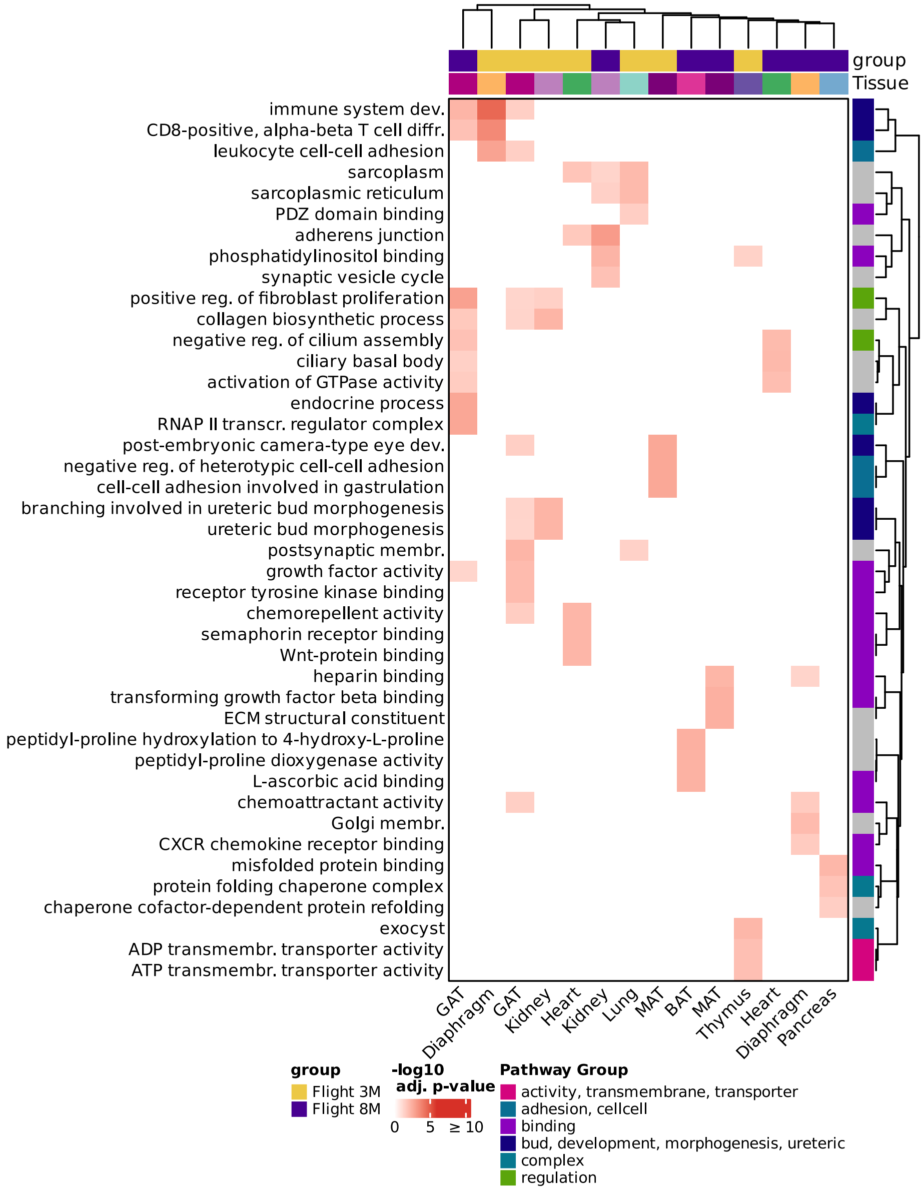

Supplementary Figure 9: Result of the Pathway analysis from 4f using validated miRNA-mRNA interactions

a. Pathway analysis as in Figure 7b using only the experimentally validated miRNA-mRNA pairs (independent of the tissue they were validated in).
