# Supplementary material for: MiRNAs shape mouse age-independent tissue adaptation to spaceflight via ECM and developmental pathways
Source: Nat Commun. 2026 Feb 5;17:1387. doi: 10.1038/s41467-026-68737-1 (PMC12876965; doi:10.1038/s41467-026-68737-1)
Supplement: Supplementary file 2 — Description of Additional Supplementary Files [file 41467_2026_68737_MOESM2_ESM.pdf]

Description of Additional Supplementary Files

File name: Supplementary Data 1

Description: Metadata of the mice.

File name: Supplementary Data 2

Description: Differentially expressed miRNAs (two-sided Wilkox Rank Sum Test; BH adjusted).

File name: Supplementary Data 3

Description: Results of the ORA Pathway Analysis (ORA, one-sided, BH adjusted).

File name: Supplementary Data 4

Description: Results of the GSEA Pathway Analysis with miEAA (GSEA, one-sided, BH adjusted).

File name: Supplementary Data 5

Description: Differentially expressed mRNAs (two-sided Wilkox Rank Sum Test; BH adjusted).

File name: Supplementary Data 6

Description: Experimentally validated mRNA-miRNA pairs (from Figure 4a).

File name: Supplementary Data 7

Description: Differentially expressed miRNAs in the female mice of the TMS cohort (two-sided Wilkox Rank Sum Test; BH adjusted).

File name: Supplementary Data 8

Description: Experimentally validated mRNA-miRNA pairs (from Figure 7a).

File name: Supplementary Data 9

Description: Overlap of the miRNAs reported in Figure 3b, 4c and 7d with circulating miRNAs from blood, plasms and serum in the miRNATissueAtlas 2025.
