# Supplementary material for: MiRNAs shape mouse age-independent tissue adaptation to spaceflight via ECM and developmental pathways
Source: Nat Commun. 2026 Feb 5;17:1387. doi: 10.1038/s41467-026-68737-1 (PMC12876965; doi:10.1038/s41467-026-68737-1)
Supplement: Supplementary file 12 — Reporting Summary [file 41467_2026_68737_MOESM12_ESM.pdf]

nature portfolio

Corresponding author(s): Andreas Keller
Last updated by author(s): Dec 19, 2025

Reporting Summary

Nature Portfolio wishes to improve the reproducibility of the work that we publish. This form provides structure for consistency and transparency in reporting. For further information on Nature Portfolio policies, see our Editorial Policies and the Editorial Policy Checklist.

Statistics

For all statistical analyses, confirm that the following items are present in the figure legend, table legend, main text, or Methods section.

n/a	Confirmed	
☐	☒	The exact sample size (*n*) for each experimental group/condition, given as a discrete number and unit of measurement
☐	☒	A statement on whether measurements were taken from distinct samples or whether the same sample was measured repeatedly
☐	☒	The statistical test(s) used AND whether they are one- or two-sided<br>*Only common tests should be described solely by name; describe more complex techniques in the Methods section.*
☐	☒	A description of all covariates tested
☐	☒	A description of any assumptions or corrections, such as tests of normality and adjustment for multiple comparisons
☐	☒	A full description of the statistical parameters including central tendency (e.g. means) or other basic estimates (e.g. regression coefficient) AND variation (e.g. standard deviation) or associated estimates of uncertainty (e.g. confidence intervals)
☐	☒	For null hypothesis testing, the test statistic (e.g. *F*, *t*, *r*) with confidence intervals, effect sizes, degrees of freedom and *P* value noted<br>*Give P values as exact values whenever suitable.*
☒	☐	For Bayesian analysis, information on the choice of priors and Markov chain Monte Carlo settings
☒	☐	For hierarchical and complex designs, identification of the appropriate level for tests and full reporting of outcomes
☐	☒	Estimates of effect sizes (e.g. Cohen's *d*, Pearson's *r*), indicating how they were calculated

Our web collection on statistics for biologists contains articles on many of the points above.

Software and code

Policy information about availability of computer code

Data collection	Fastq files containing raw sequencing reads were mapped against GRCm39 and miRBase (version 22.1) 65 in order to profile miRNA counts using miRmaster (version 2.0). Information about miRNA family was obtained from mirgenedb 3.0.
Data analysis	Custom scripts used to produce the figures of this study are available from github (http://doi.org/10.5281/zenodo.17989911). The following pachages and databases were used for the study: mirmaster v2.0, umap-learn v0.3.10, Cellranger v5.0.0, SoupX v1.5.2, Seurat v4.0.3, DoubletFinder v2.0.3, GeneTrail v3.2, muscat v1.12.0, mieaa v2.0, clusterProfiler v4.6.0, OntologyIndex v2.12, org.Mm.eg.db v3.16.0, simplifyEnrichment v1.8.0, miRNATissueAtlas2025, TargetScan Release 8.0, miRTarBase 2025, GRCm3, Python v3.12.0, scanpy v1.10.2, scikit-learn v1.5.1, scipy v1.14.0, seaborn v0.13.2, matplotlib-base v3.9.1

For manuscripts utilizing custom algorithms or software that are central to the research but not yet described in published literature, software must be made available to editors and reviewers. We strongly encourage code deposition in a community repository (e.g. GitHub). See the Nature Portfolio guidelines for submitting code & software for further information.

Data

Policy information about availability of data

All manuscripts must include a data availability statement. This statement should provide the following information, where applicable:

- Accession codes, unique identifiers, or web links for publicly available datasets
- A description of any restrictions on data availability
- For clinical datasets or third party data, please ensure that the statement adheres to our policy

The raw and processed miRNA data have been deposited at GEO under accession code GSE294046 [https://www.ncbi.nlm.nih.gov/geo/query/acc.cgi?acc=GSE294046]. The raw and processed mRNA data have been deposited at GEO under accession code GSE295428 [https://www.ncbi.nlm.nih.gov/geo/query/acc.cgi?acc=GSE295428]. The mRNA and the miRNA data have furthermore been deposited at and at NASE OSDR under accessions OSD-904 [http://doi.org/10.26030/ftkn-c363], OSD-905 [http://doi.org/10.26030/578r-zb36], OSD-906 [https://doi.org/10.26030/32kg-hg35], OSD-907 [http://doi.org/10.26030/h2qz-2x82], OSD-908 [http://doi.org/10.26030/fhxa-pp75], OSD-909 [http://doi.org/10.26030/bnfb-5953], OSD-910 [http://doi.org/10.26030/rybg-df97], OSD-911 [http://doi.org/10.26030/e9n1-9b95], OSD-912 [http://doi.org/10.26030/yrv8-d312], OSD-913 [http://doi.org/10.26030/t6vh-cg75], OSD-914 [http://doi.org/10.26030/g6aq-7884], OSD-915 [http://doi.org/10.26030/kpns-6s32], OSD-916 [http://doi.org/10.26030/bfs0-4m24], . Source Data are provided with this paper.

Research involving human participants, their data, or biological material

Policy information about studies with human participants or human data. See also policy information about sex, gender (identity/presentation), and sexual orientation and race, ethnicity and racism.

Reporting on sex and gender	*Use the terms sex (biological attribute) and gender (shaped by social and cultural circumstances) carefully in order to avoid confusing both terms. Indicate if findings apply to only one sex or gender; describe whether sex and gender were considered in study design; whether sex and/or gender was determined based on self-reporting or assigned and methods used.* *Provide in the source data disaggregated sex and gender data, where this information has been collected, and if consent has been obtained for sharing of individual-level data; provide overall numbers in this Reporting Summary. Please state if this information has not been collected.* *Report sex- and gender-based analyses where performed, justify reasons for lack of sex- and gender-based analysis.*
Reporting on race, ethnicity, or other socially relevant groupings	*Please specify the socially constructed or socially relevant categorization variable(s) used in your manuscript and explain why they were used. Please note that such variables should not be used as proxies for other socially constructed/relevant variables (for example, race or ethnicity should not be used as a proxy for socioeconomic status).* *Provide clear definitions of the relevant terms used, how they were provided (by the participants/respondents, the researchers, or third parties), and the method(s) used to classify people into the different categories (e.g. self-report, census or administrative data, social media data, etc.)* *Please provide details about how you controlled for confounding variables in your analyses.*
Population characteristics	*Describe the covariate-relevant population characteristics of the human research participants (e.g. age, genotypic information, past and current diagnosis and treatment categories). If you filled out the behavioural & social sciences study design questions and have nothing to add here, write "See above."*
Recruitment	*Describe how participants were recruited. Outline any potential self-selection bias or other biases that may be present and how these are likely to impact results.*
Ethics oversight	*Identify the organization(s) that approved the study protocol.*

Note that full information on the approval of the study protocol must also be provided in the manuscript.

Field-specific reporting

Please select the one below that is the best fit for your research. If you are not sure, read the appropriate sections before making your selection.

- [x] Life sciences
- [ ] Behavioural & social sciences
- [ ] Ecological, evolutionary & environmental sciences

For a reference copy of the document with all sections, see nature.com/documents/nr-reporting-summary-flat.pdf

Life sciences study design

All studies must disclose on these points even when the disclosure is negative.

Sample size	No statistical method was used to predetermine sample size. For the miRNA analyses, all available mice from each experimental group were included (see Samples and Cohorts). No data were excluded from the analyses. The experiments were not randomized, and the investigators were not blinded to group allocation during sample collection, processing, or outcome assessment.
Data exclusions	Biological replicates included in this study were successfully processed and yielded usable data, with the exception of a small number of samples that were excluded during pre-processing (detailed threshold see Methods). Technical replicates performed during RNA extraction and library preparation met quality control standards. No other failures occurred during sample processing or sequencing that would prevent replication of the experiments described.

Replication	For this dataset, we used 40 mice per group (4 from Fl (LAR) and 4 from HGC (LAR) were used for the mRNA experiment). We extracted multiple tissues from each mouse (exact number see Supplementary Data 1).
Randomization	All mice were female, so sex was not a confounding variable. Age was accounted for by grouping mice into young (3 months) and old (8 months) categories in all analyses. Ground controls were matched to Flight mice for housing conditions (temperature, humidity, light/dark cycle, diet, and habitat density). Tissue collection was staggered across groups to minimize circadian or procedural effects. RNA extraction, library preparation, and sequencing were randomized across plates and runs to reduce technical variation. Potential confounders, such as stress due to spaceflight or delayed return of Flight mice, were recorded and considered in the interpretation of results. No additional covariates were explicitly controlled.
Blinding	Blinding was not relevant for this study. This study is observational and study outcomes were quantitative sequencing measurements rather than subjective assessments

Reporting for specific materials, systems and methods

We require information from authors about some types of materials, experimental systems and methods used in many studies. Here, indicate whether each material, system or method listed is relevant to your study. If you are not sure if a list item applies to your research, read the appropriate section before selecting a response.

Materials & experimental systems

n/a	Involved in the study
x	Antibodies
x	Eukaryotic cell lines
x	Palaeontology and archaeology
	x Animals and other organisms
x	Clinical data
x	Dual use research of concern
x	Plants

Methods

n/a	Involved in the study
x	ChIP-seq
x	Flow cytometry
x	MRI-based neuroimaging

Animals and other research organisms

Policy information about studies involving animals; ARRIVE guidelines recommended for reporting animal research, and Sex and Gender in Research

Laboratory animals	All mice were BALB/cAnNTac female mice (Taconic Biosciences). Mice were grouped into two age categories at the start of the experiment: 'young' (~3 months) and 'old' (~8 months). Exact ages of individual mice were not recorded; these groups represent standard age categories used in Rodent Research ISS missions.
Wild animals	No wild animals were included in this study
Reporting on sex	All mice were female. Sex-specific analysis was not possible due to the lack of male mice.
Field-collected samples	VGC mice were housed at NASA Kennedy Space Center in standard vivarium conditions at 4 mice per cage, 20-22.2 °C, 12hr light 12hr dark. HGC mice housing was matched to the conditions experienced by Flight mice (as closely as possible) by using double density housing (the same Rodent Habitats (AEM-X) and Animal Enclosure Module Transporters (AEM-T) as the flight animals), with identical food, matched temperature, humidity, and carbon dioxide levels based on actual flight environmental monitoring. Each habitat contained ten mice (five per side) and included enrichment huts as part of the AEM-X configuration. To dissect all mice together in matching conditions, VGC and HGC mice were transported to Scripps Institute just before the return of the Flight mice and were dissected in matching conditions (+/- 1 day).
Ethics oversight	This experiment was approved by the NASA Flight Animal Care and Use Committee (Protocol #FLT-18-116) at the NASA Ames Research Center (Moffett Field, CA) and the Kennedy Space Center (KSC, FL). The Flight mice were received after landing at Scripps Research Institute (09-0004, for animal receipt upon landing) and transported to Stanford University IACUC (WYS1591).

Note that full information on the approval of the study protocol must also be provided in the manuscript.

Plants

Seed stocks	*Report on the source of all seed stocks or other plant material used. If applicable, state the seed stock centre and catalogue number. If plant specimens were collected from the field, describe the collection location, date and sampling procedures.*
Novel plant genotypes	*Describe the methods by which all novel plant genotypes were produced. This includes those generated by transgenic approaches, gene editing, chemical/radiation-based mutagenesis and hybridization. For transgenic lines, describe the transformation method, the number of independent lines analyzed and the generation upon which experiments were performed. For gene-edited lines, describe the editor used, the endogenous sequence targeted for editing, the targeting guide RNA sequence (if applicable) and how the editor was applied.*
Authentication	*Describe any authentication procedures for each seed stock used or novel genotype generated. Describe any experiments used to assess the effect of a mutation and, where applicable, how potential secondary effects (e.g. second site T-DNA insertions, mosiacism, off-target gene editing) were examined.*
